# Preliminary Contrast-Enhanced CT Imaging of the Coelom in an Adult Male African White-Backed Vulture (*Gyps africanus*): A Case Study Using Maximum Intensity Projection and Three-Dimensional Volume Rendering

**DOI:** 10.3390/ani16193079

**Published:** 2026-09-30

**Authors:** José Perera, Mario Encinoso, Benedetta Marrone, Jezlia Bolaños, Alberto Arencibia

**Affiliations:** 1 Hospital Clínico Veterinario, Facultad de Veterinaria, Universidad de Las Palmas de Gran Canaria, 35413 Arucas, Las Palmas, Spain; jose.perera@fpct.ulpgc.es (J.P.); mario.encinoso@ulpgc.es (M.E.); 2 Oasis Wildlife Fuerteventura, Museo del Campo Majorero, Carretera General Jandía, s/n, La Lajita, 35627 Pájara, Las Palmas, Spain; veterinarios@oasiswildlifefuerteventura.com; 3 Departamento de Morfología, Facultad de Veterinaria, Universidad de Las Palmas de Gran Canaria, 35413 Arucas, Las Palmas, Spain; jezlia.bolanos101@alu.ulpgc.es

**Keywords:** contrast-enhanced computed tomography, avian anatomy, coelom, *Gyps africanus*

## Abstract

Cross-sectional imaging data for the African white-backed vulture (*Gyps africanus*) are lacking, and interpretation relies mainly on general avian descriptions and studies in other birds. This preliminary case study evaluated contrast-enhanced computed tomography (CT) for visualising principal coelom structures and their spatial relationships in an adult male *G. africanus*. Images were assessed using multiplanar and three-dimensional (3D) reconstruction techniques. Soft-tissue images better depicted parenchymal organs and cardiovascular structures, whereas lung-window images improved visualisation of the respiratory system, air sacs, and pneumatised skeletal regions. Maximum intensity projection (MIP) and 3D volume rendering (VR) provided complementary information on vascular anatomy and spatial relationships. The observed anatomical features were generally consistent with established avian anatomy and previous imaging studies in other species. CT provided useful anatomical information in this individual and may support future comparative and clinical imaging studies in *G. africanus*. However, because only one bird was examined and no complementary anatomical sectioning or dissection was performed, the findings should be regarded as a preliminary case study rather than species-specific anatomical reference data.

## Simple Summary

The African white-backed vulture (*Gyps africanus*) is a critically endangered species, and a better understanding of its anatomy may help improve veterinary assessment and management of birds under human care. This preliminary case study used contrast-enhanced computed tomography (CT) to describe the main organs and blood vessels within the body cavity of an adult male *G. africanus*. CT imaging allowed these structures to be clearly identified and their position and relationships with surrounding organs to be assessed. The use of contrast medium was particularly valuable for visualising the vascular system. Although the study was based on a single case, the findings provide preliminary CT anatomical observations that may support future imaging studies in this species. This work may help veterinarians, biologists, and researchers interpret CT examinations and support future anatomical and diagnostic imaging studies in vultures and other avian species.

## 1. Introduction

The African white-backed vulture (*Gyps africanus*) is a large Old-World vulture of the family Accipitridae and one of the most widely distributed vulture species in sub-Saharan Africa [1,2,3]. Despite its historically wide distribution, populations have declined markedly across much of its range in recent decades [3,4,5,6], leading to its classification as critically endangered on the IUCN Red List [3]. Major threats include habitat degradation, poisoning, exposure to toxic contaminants, and electrocution or collision with power infrastructure [3,5,6,7,8,9]. Consequently, injured or debilitated individuals may be admitted to wildlife rehabilitation centres, where they undergo clinical assessment, treatment, and rehabilitation [10].

The anatomy of vultures reflects a high degree of functional specialisation associated with flight and scavenging. *G. africanus* possesses extensive skeletal pneumaticity and morphological adaptations related to efficient soaring flight [11]. In addition, the distinctive organisation of the avian coelom results in complex spatial relationships among the viscera [12,13,14,15]. Unlike the separate thoracic and abdominal cavities of mammals, the avian coelom exhibits a highly specialised compartmental organisation, and some organs, including the kidneys and adrenal glands, are located retroperitoneally [12,13,14,15]. Knowledge of these anatomical relationships is essential for the accurate interpretation of cross-sectional diagnostic images [16,17,18,19,20,21,22,23,24].

Computed tomography (CT) is an important imaging modality in avian medicine and anatomical research, providing detailed cross-sectional images that can be reconstructed in multiple planes and used for three-dimensional (3D) visualisation of complex anatomical structures [16,17,18,19,20,21,22,23,24,25,26,27].

In birds, CT is particularly useful for evaluating coelom organs because it avoids anatomical superimposition inherent to conventional radiography and enables a more precise assessment of their spatial relationships [16,17,19,21]. This approach may further improve soft-tissue differentiation and anatomical delineation and may facilitate evaluation of vascular structures and organ enhancement [18,20,24].

Advanced post-processing techniques, including maximum intensity projection (MIP) and 3D volume rendering (VR), can further improve the visualisation of spatial relationships among vascular, skeletal, and soft-tissue structures [24,25,26,27].

Although CT-based anatomical descriptions have been reported in several avian species [17,18,19,20,21,22,23,24,25], no published CT anatomical studies appear to be available for large African vultures. To the best of the authors’ knowledge, no previous study has described the contrast-enhanced CT anatomy of the coelom in *G. africanus*. Accordingly, assessment of this species currently relies largely on general avian anatomical descriptions and information derived from other avian taxa. Preliminary imaging observations in *G. africanus* may help determine the extent to which these general anatomical patterns are applicable to this species and provide a basis for future comparative and clinical imaging studies.

Therefore, the aim of the present study was to provide a preliminary contrast-enhanced CT case study of the coelom of an adult male *G. africanus*. These images, together with MIP and 3D VR reconstructions, were used to characterise the coelom viscera, major vascular structures, and their spatial relationships.

## 2. Materials and Methods

### 2.1. Animals

A captive adult male *G. africanus* resident at Oasis Wildlife Fuerteventura (Spain) was examined for this study. At the time of imaging, the bird was 22 years old and had a body weight of 3.5 kg. Permission to perform the examination was obtained from the animal’s owner, and all procedures were carried out in compliance with the applicable animal-welfare regulations. Veterinary professionals were responsible for conducting all diagnostic procedures, and appropriate welfare measures were maintained at all stages of the procedure.

### 2.2. Anaesthesia and Monitoring of Physiological Parameters

Anaesthesia was initiated by placing the vulture in an 80 cm × 60 cm × 60 cm induction chamber supplied with 4% sevoflurane (SevoFlo; Abbott Laboratories SA, Madrid, Spain) in oxygen. This method was chosen to reduce the need for physical restraint during induction and thereby limit handling-associated stress in this large raptor. Once an adequate plane of anaesthesia had been achieved, the bird was removed from the chamber and anaesthesia was continued using 2% sevoflurane in oxygen, delivered through a face mask at a flow rate of 2.0 L/min. Spontaneous respiratory movements were assessed visually, aided by the placement of a small gauze swab over the thoracic region.

Cardiorespiratory monitoring included continuous assessment of heart rate, respiratory rate and peripheral oxygen saturation by pulse oximetry in conjunction with direct clinical observation. Measures to reduce peri-anaesthetic heat loss included the use of warming blankets and an increase in ambient room temperature from 20 °C to 25 °C, with the aim of maintaining thermal stability during the procedure.

### 2.3. Contrast Medium Administration and Contrast-Enhanced CT Acquisition

For CT examination, the vulture was placed in dorsal recumbency and positioned symmetrically with the longitudinal axis of the body aligned in a craniocaudal direction. Image acquisition was performed with a 16-slice helical CT system (Toshiba Astelion; Canon Medical Systems, Tokyo, Japan). The scanning protocol comprised a tube voltage of 100 kVp, a tube current–time product of 50 mAs, a slice thickness of 1 mm and a reconstruction interval of 1 mm. A 512 × 512 image matrix was used for reconstruction, with a helical pitch of 0.93 and a gantry rotation time of 0.75 s.

To enhance differentiation of the coelom viscera and improve depiction of the vascular structures, a non-ionic iodinated contrast agent (iobitridol; Xenetix^®^, 300 mg I/mL; Guerbet, Villepinte, France) was administered intravenously at a dose of 400 mg I/kg. Vascular access was obtained using a 22-gauge catheter inserted into the right brachial vein. Contrast administration was undertaken under veterinary supervision as part of the anaesthetic and imaging procedure, while the physiological status of the bird was monitored continuously.

Once CT acquisition had been completed, the vulture was moved to a temperature-controlled area for recovery from anaesthesia. Clinical monitoring was continued during the recovery period until the bird had regained full consciousness and exhibited normal behavioural activity.

### 2.4. Contrast-Enhanced CT Image Reconstruction, Post-Processing, and Analysis

Following CT acquisition, the complete image datasets were preserved in their original form and exported in Digital Imaging and Communications in Medicine (DICOM) format to a dedicated image-processing workstation equipped with OsiriX MD (version 13.0.2; Pixmeo, Bernex, Switzerland). All subsequent image processing, reconstruction and anatomical evaluation were carried out using this software.

For image interpretation, the CT series were reviewed using two predefined display settings selected to optimise visualisation of the principal structures within the coelom. Soft-tissue structures were primarily assessed using a window width (WW) of 350 and a window level (WL) of 40. A lung window (WW, 1400; WL, −500) was additionally applied to improve assessment of air-containing structures and anatomical interfaces adjacent to aerated regions. The two window settings were used as appropriate according to the anatomical region and structures under evaluation.

MPR images were generated from the original CT volume in the transverse and sagittal planes to facilitate assessment of the topographical relationships among coelom structures. MIP reconstructions were subsequently produced using the multiplanar datasets, with particular emphasis on enhancing depiction of contrast-opacified vessels and other structures showing relatively high attenuation. These reconstructions provided an additional means of evaluating the course, distribution and spatial relationships of the major vascular structures with adjacent coelomic organs.

3D VR reconstructions were subsequently generated from the CT datasets to provide a more intuitive representation of the spatial arrangement of the principal coelom structures. Prior to rendering, osseous structures and other tissues that were not relevant to the intended anatomical assessment were manually removed from the datasets to reduce visual overlap and improve delineation of the structures of interest. The resulting 3D VR images were then examined from different viewing perspectives to facilitate interpretation of the 3D relationships among the coelom organs and associated vasculature. All source CT images, MIP reconstructions and VR datasets were evaluated under standardised viewing conditions to ensure consistency throughout the anatomical assessment.

For preparation of the figures, representative CT images were selected and processed using Adobe Photoshop CS5 (Adobe Systems Inc., San Jose, CA, USA). Image editing was limited to minor adjustments in contrast and sharpness intended solely to improve clarity and overall visual presentation. No modifications were made that altered, removed or introduced anatomical features, and the diagnostic and anatomical content of the original images was preserved.

Coelom structures identified on the contrast-enhanced CT images were evaluated systematically according to their anatomical position, overall morphology, relationship to neighbouring organs and vessels, and characteristic attenuation and enhancement features. Attention was paid to the topographical arrangement of the structures within the coelom and to their spatial relationships with adjacent anatomical landmarks. Identification and labelling were performed using established avian anatomical sources as the principal reference framework [12,13,14,15], with anatomical terminology applied consistently throughout the text, figure annotations, and supplementary table, in accordance with the Nomina Anatomica Avium [13].

## 3. Results

Contrast-enhanced CT images were initially assessed using transverse and sagittal MIP reconstructions displayed with both soft-tissue and lung window settings, enabling detailed evaluation of the principal osseous, muscular, respiratory, cardiovascular and visceral structures of the coelom in the adult male *G. africanus*. The two MIP window settings provided complementary anatomical information. Soft-tissue window images afforded greater differentiation of the musculature, cardiovascular structures and parenchymal organs, whereas lung-window images provided superior visualisation of the respiratory tract, air-sac system and pneumatised skeletal structures.

In Figure 1, a standard sagittal reference image (A) indicates the approximate craniocaudal levels corresponding to the transverse MIP reconstructions shown in Figure 2, Figure 3, Figure 4, Figure 5, Figure 6, Figure 7, Figure 8 and Figure 9, while a standard transverse reference image (B) indicates the approximate locations of the two sagittal MIP planes presented in Figure 10 and Figure 11.

These transverse reconstructions were arranged sequentially in a craniocaudal direction, extending from the level of the right and left cranial venae cavae to the caudal division of the kidneys and encompassing representative anatomical levels throughout the coelom. At each transverse level, paired MIP images were displayed using (A) soft-tissue and (B) lung window settings, allowing direct comparison of the musculoskeletal structures, soft tissues, pulmonary parenchyma, air sacs, skeletal pneumatisation and vasculature. Sagittal reconstructions were arranged sequentially from right to left, extending from the level of the ascending aorta and aortic arch to the proventriculus and ventriculus. As in the transverse series, each sagittal plane was represented by paired MIP images displayed using (A) soft-tissue and (B) lung window settings, facilitating direct comparison of the principal musculoskeletal, pulmonary, visceral and vascular structures.

The coelom exhibited a complex compartmentalised organisation, with the visceral organs and air-sac system occupying distinct but closely related regions. These structures were arranged along both the craniocaudal and dorsoventral axes and maintained characteristic topographical relationships with neighbouring viscera, the body wall, the air sacs, and adjacent pneumatised bones. This organisation provided the anatomical framework for evaluation of the different coelom systems on the transverse and sagittal MIP reconstructions.

The vertebral column, ribs, sternum and synsacrum served as the principal osseous landmarks for spatial orientation using skeletotopy, while the surrounding body-wall musculature defined the external boundaries of the coelom. Cortical bone exhibited high attenuation on soft-tissue window MIP images, whereas bone marrow showed intermediate soft-tissue attenuation within non-pneumatised skeletal regions. In contrast, pneumatised areas of the axial and appendicular skeleton contained air-filled regions of markedly lower attenuation. These air-containing skeletal regions were more clearly delineated on lung-window MIP images, particularly within the vertebral column, synsacrum and proximal portions of the limb girdles. Bone marrow was more readily differentiated from the surrounding cortical bone on soft-tissue window MIP images, while its conspicuity was reduced on lung-window images. The musculature displayed relatively homogeneous intermediate attenuation and could be readily distinguished from adjacent soft tissues using the soft-tissue window. Although the muscular structures remained identifiable on lung-window images, soft-tissue contrast and internal muscular detail were reduced.

The heart and associated major vessels occupied the cranial portion of the coelom. The myocardium demonstrated intermediate soft-tissue attenuation and was most clearly delineated on soft-tissue window MIP images. Following intravenous contrast administration, the atrial and ventricular chambers became readily distinguishable, while the lumina of the principal blood vessels showed marked and relatively homogeneous enhancement. The major cardiac and vascular structures remained recognisable on lung-window images, although myocardial detail and vascular margins were less conspicuous than with the soft-tissue window. The right atrium received systemic venous return from the paired right and left cranial venae cavae and the caudal vena cava (Figure 2, Figure 4, Figure 5, Figure 6 and Figure 10), whereas the left atrium received the pulmonary veins (Figure 3, Figure 4, Figure 10 and Figure 11). The pulmonary trunk and its right and left pulmonary arteries were also identified (Figure 3, Figure 4, Figure 10 and Figure 11). Additional major vessels included the brachiocephalic trunk (Figure 2), the ascending aorta (Figure 3 and Figure 10) and the descending aorta (Figure 3, Figure 4, Figure 5, Figure 6, Figure 7, Figure 8, Figure 9 and Figure 11). The external jugular veins joined the corresponding brachiocephalic veins before draining into the paired right and left cranial venae cavae (Figure 2 and Figure 10).

The respiratory tract could be followed from the trachea to the intrapulmonary bronchial system. Caudally, the trachea terminated at the syrinx, from which the right and left principal bronchi arose and continued towards the lungs. The lungs were bilaterally symmetrical, positioned in close contact with the dorsal body wall, and displayed the compact, non-expansile configuration characteristic of the avian respiratory system. Visceral pleura covered the pulmonary surfaces, while parietal pleura lined the adjacent body wall. The pleural space was extremely narrow and could not be individually discerned on contrast-enhanced CT images. At the pulmonary hila, the principal bronchi entered the pulmonary parenchyma and continued as the intrapulmonary bronchial system. Smaller bronchial branches could also be recognised within the pulmonary tissue, although their individual anatomical identities could not be confidently established on CT. On soft-tissue window MIP images, the air-containing components of the respiratory system were poorly delineated, and the pulmonary parenchyma was only faintly appreciable (Figure 2A, Figure 3A, Figure 4A, Figure 5A and Figure 10A). By contrast, lung-window MIP reconstructions provided substantially greater definition. The trachea, syrinx, principal bronchi and visible intrapulmonary branches appeared as low-attenuation tubular structures bordered by thin, soft-tissue walls. The pulmonary parenchyma was also more clearly depicted on lung-window images, showing relatively low attenuation, smooth external contours and a fine internal pattern consistent with aerated pulmonary tissue (Figure 2B, Figure 3B, Figure 4B, Figure 5B and Figure 10B). However, individual parabronchi could not be confidently distinguished because of the spatial resolution achieved with the CT protocol used.

The air-sac system formed an extensive network of thin-walled, air-filled compartments distributed throughout much of the coelom and closely associated with the surrounding viscera, body wall and pneumatised skeleton. Individual sacs were recognised primarily according to their characteristic topographical position and anatomical relationships. The system comprised paired cervical, cranial thoracic, caudal thoracic and abdominal air sacs, together with an unpaired clavicular air sac. The cervical air sacs communicated with vertebral diverticula, whereas the clavicular, or interclavicular, air sac gave rise to sternal, subpectoral, subscapular, axillary and humeral diverticula. The cranial and caudal thoracic air sacs were situated ventral to the lungs and showed a distinct craniocaudal distribution. The cranial thoracic sacs occupied the more cranial region of the coelom and extended caudally along the body wall, whereas the caudal thoracic sacs were positioned further caudally and were closely related to the abdominal air sacs. The paired abdominal air sacs extended throughout the caudal coelom and formed diverticular extensions associated with the pelvic, perirenal and femoral regions. Collectively, the air sacs and their diverticula constituted an extensive interconnected pneumatic system extending through much of the coelom and into several pneumatised regions of the skeleton.

The air-sac walls were generally not distinguishable on CT, although short, faint wall segments could occasionally be appreciated. No wall thickening or intraluminal material was identified. Although the principal air sacs could be recognised, the interfaces between adjacent sacs could not be consistently followed throughout their entire extent. Consequently, individual sacs and diverticula were identified only when their position and relationships with neighbouring anatomical structures permitted sufficiently confident recognition. On soft-tissue window MIP images, the air sacs were poorly conspicuous, and their boundaries were difficult to distinguish from neighbouring structures. Lung-window MIP images provided markedly improved visualisation of these air-filled compartments and their diverticular extensions, allowing their distribution and relationships with the surrounding viscera, body wall and pneumatised skeletal structures to be assessed more clearly (Figure 2B, Figure 3B, Figure 4B, Figure 5B, Figure 6B, Figure 7B, Figure 8B, Figure 9B, Figure 10B and Figure 11B).

The gastrointestinal tract was located predominantly within the ventral aspect of the coelom and followed a generally craniocaudal course. Within the coelom examined, the oesophagus continued caudally into the proventriculus, which in turn continued into the ventriculus. The ingluvies, located in the cervical region, was outside the anatomical scope of the present coelomic study. From the ventriculus, the intestinal tract extended as multiple loops, most of which were contained within the common intestinal peritoneal cavity. Although the intestinal loops could be visualised, none of the individual intestinal segments, including the duodenum, jejunum, ileum, caeca, and rectum, could be differentiated with sufficient confidence on the CT images.

On soft-tissue window MIP images, the gastrointestinal wall exhibited intermediate attenuation and could generally be differentiated from the lower-attenuation luminal contents (Figure 2A, Figure 3A, Figure 4A, Figure 5A, Figure 6A, Figure 7A, Figure 8A, Figure 9A, Figure 10A and Figure 11A). This distinction was reduced on lung-window images (Figure 2B, Figure 3B, Figure 4B, Figure 5B, Figure 6B, Figure 7B, Figure 8B, Figure 9B, Figure 10B and Figure 11B), resulting in less precise delineation of the gastrointestinal tract.

The liver occupied the cranial portion of the coelom immediately caudal to the heart and extended to either side of the midline. Two well-defined hepatic lobes were recognised on CT. On soft-tissue window MIP images, the hepatic parenchyma appeared relatively homogeneous and displayed generally uniform enhancement following intravenous contrast administration. The hepatic veins were readily identifiable as contrast-enhanced vascular channels coursing through the liver (Figure 5A, Figure 6A, Figure 7A, Figure 10A and Figure 11A). The gallbladder was closely associated with the hepatic parenchyma and appeared as a thin-walled, pear-shaped structure containing material of lower attenuation than the surrounding liver. Its boundaries were most clearly appreciated on soft-tissue window images (Figure 6A, Figure 7A and Figure 10A). On lung-window MIP images, hepatic soft-tissue differentiation was reduced (Figure 5B, Figure 6B, Figure 7B, Figure 10B and Figure 11B), and the margins of the gallbladder were consequently less distinct (Figure 6B, Figure 7B and Figure 10B). Nevertheless, its lower attenuation relative to the adjacent hepatic parenchyma still permitted recognition. The excretory bile ducts could not be confidently visualised under the conditions of this CT examination. Although the right and left hepatic portal veins could not be individually differentiated with sufficient confidence in most images, the hepatic portal veins were clearly and correctly visualised in Figure 6 and Figure 10.

The spleen was situated in close apposition to the proventriculus, which provided a consistent anatomical landmark for its identification. It appeared as a well-demarcated soft-tissue structure showing relatively homogeneous attenuation and contrast enhancement. Soft-tissue window MIP images provided the clearest definition of the splenic parenchyma and margins (Figure 6A, Figure 10A and Figure 11A), enabling reliable differentiation from adjacent viscera. The spleen remained recognisable on lung-window images, although its boundaries were less conspicuous because of the reduced soft-tissue contrast (Figure 6B, Figure 10B and Figure 11B). In contrast to the other parenchymal organs, the pancreas could not be reliably identified with either MIP window setting. Its margins could not be confidently differentiated from the surrounding coelom soft tissues, particularly in the region of the duodenal loop. No discrete pancreatic margins or internal features could be consistently distinguished from the adjacent soft tissues under the imaging conditions used.

Several additional major vessels were identified within the central and caudal portions of the coelom. The right and left common iliac veins converged caudally to form the caudal vena cava, which subsequently coursed cranially through the coelom. The cranial and caudal mesenteric veins were also visualised and united to form a common mesenteric vein before entering the hepatic portal circulation. Intravenous administration of contrast medium markedly increased vascular attenuation and thereby improved differentiation of the vascular lumina from the surrounding soft tissues. Although these vessels could be identified using both MIP window settings, their margins and course were more clearly demonstrated on soft-tissue window images owing to the greater contrast between the enhanced vascular lumen and adjacent tissues (Figure 6A, Figure 7A, Figure 8A, Figure 9A and Figure 10A).

The paired kidneys occupied a retroperitoneal position within the renal fossae of the synsacrum and were closely moulded against the adjacent synsacral bone, reflecting the intimate association of the avian renal system with the pelvic region. Each kidney could be divided into cranial, middle and caudal divisions. On soft-tissue window MIP images, the renal parenchyma displayed relatively homogeneous attenuation, preservation of its gross internal organisation and a generally uniform enhancement pattern following intravenous contrast administration (Figure 6A, Figure 7A, Figure 8A, Figure 9A, Figure 10A and Figure 11A). The kidneys remained identifiable on lung-window MIP images, predominantly owing to their characteristic location and overall attenuation (Figure 6B, Figure 7B, Figure 8B, Figure 9B, Figure 10B and Figure 11B). However, their margins and internal parenchymal architecture were less conspicuous than on soft-tissue window images.

From each kidney, the ureters coursed caudally along the dorsal aspect of the coelom before reaching the cloaca. Renal and pelvic segments could be distinguished along their course, progressively converging caudally until the pelvic segment entered the urodeum. Following intravenous contrast administration, the ureters appeared as slender tubular structures showing mildly increased attenuation. On soft-tissue window MIP images, this increase in attenuation facilitated delineation of the ureteral lumen and differentiation from the surrounding soft tissues (Figure 6A, Figure 7A, Figure 9A and Figure 10A). The ureters could also be recognised on lung-window MIP images (Figure 6B, Figure 7B, Figure 9B and Figure 10B), although their attenuation and margins were less distinct than with the soft-tissue window.

The paired adrenal glands were situated craniomedial to the cranial divisions of the kidneys and were closely associated with the renal region. They appeared as small elongated-to-ovoid structures displaying relatively homogeneous soft-tissue attenuation and subtle margins. On soft-tissue window MIP images, their location, contour and attenuation could be assessed more clearly, although distinction from the adjacent renal parenchyma remained limited (Figure 7A). On lung-window images, the adrenal glands were less conspicuous because of the lower soft-tissue contrast, making their margins more difficult to distinguish from neighbouring structures (Figure 7B).

Paired testes were identified in a dorsolateral position within the coelom, adjacent to the cranial divisions of the kidneys. Both testes were small and relatively homogeneous, with comparable morphology, attenuation and contrast enhancement. On soft-tissue window MIP images, the testes were more clearly delineated from the surrounding tissues, although their internal architecture remained only faintly appreciable (Figure 6A, Figure 7A and Figure 11A). They could also be recognised on lung-window MIP images according to their characteristic location and overall attenuation; however, their margins and internal structural detail were less conspicuous (Figure 6B, Figure 7B and Figure 11B) than with the soft-tissue window.

Peripheral nervous structures assessed during image interpretation could not be consistently distinguished with sufficient confidence on CT and were therefore excluded from the anatomical characterisation. No continuous neural structures could be reliably traced or differentiated from the adjacent soft tissues under the imaging conditions used. These limitations likely reflected both intrinsic anatomical factors, including the small size and close anatomical relationships of the pancreas and peripheral neural structures, and technical factors related to spatial resolution and limited soft tissue contrast under the CT protocol used.

Overall, the paired soft-tissue and lung window settings applied to the transverse and sagittal MIP reconstructions provided complementary anatomical information. Soft-tissue window MIP images afforded the greatest detail for the myocardium, contrast-enhanced vasculature, gastrointestinal wall and parenchymal organs, particularly the liver, spleen and kidneys, and provided clearer assessment of the ureters, adrenal glands and testes. Lung-window MIP images, in contrast, provided superior visualisation of the pulmonary parenchyma, tracheobronchial system, air sacs and skeletal pneumatisation. The MIP series therefore enabled detailed assessment of anatomical morphology and topographical relationships in both the transverse and sagittal planes.

Right- and left-lateral 3D VR reconstructions were evaluated as complementary representations of the coelom anatomy (Figure 12 and Figure 13, respectively). Unlike the MIP reconstructions, which enabled detailed assessment according to the selected window setting and reconstruction plane, the 3D VR images provided an integrated 3D overview of the principal visceral and vascular structures and their spatial relationships within the coelom. Partial transparency enabled structures located at different depths to be displayed simultaneously, allowing the relative positions of the major visceral organs and associated vasculature to be assessed within a single 3D view (Figure 12 and Figure 13). The rendered appearance of individual structures was primarily determined by their attenuation and the opacity settings applied during reconstruction. Contrast-enhanced vascular structures exhibited high opacity, whereas tissues of intermediate attenuation displayed varying degrees of translucency. This differential representation allowed overlapping anatomical structures to remain partially visible while reducing obscuration of deeper structures.

The cardiovascular system was particularly conspicuous on the 3D VR reconstructions. The heart and principal blood vessels, including the right and left cranial venae cavae, the caudal vena cava, the pulmonary vessels, the brachiocephalic trunks, and the aorta, were readily delineated owing to the marked increase in vascular attenuation following intravenous contrast administration and their characteristic anatomical distribution (Figure 12 and Figure 13).

Major vessels associated with the gastrointestinal and hepatic portal systems were also represented, including the cranial and caudal mesenteric veins, the common mesenteric vein, the right and left hepatic portal veins, and the hepatic veins. 3D visualisation facilitated assessment of the course of these vessels and their spatial relationships with the heart, liver, gastrointestinal tract and adjacent viscera.

Selective adjustment of opacity and attenuation ranges permitted focused visualisation of individual anatomical components. In particular, the contrast-enhanced systemic and pulmonary vessels, together with the principal mesenteric and hepatic portal veins, could be displayed in conjunction with the major visceral organs while reducing superimposition from surrounding tissues, thereby facilitating assessment of their 3D anatomical relationships (Figure 12 and Figure 13).

Among the parenchymal organs, the liver, spleen and kidneys were clearly represented on the 3D VR reconstructions (Figure 12 and Figure 13). Their overall configuration, position within the coelom and relationships with neighbouring vascular structures could therefore be assessed simultaneously. In particular, the relationships between the liver and the hepatic and mesenteric venous systems could be appreciated in three dimensions.

The pulmonary parenchyma, air-sac system and gastrointestinal tract were not visualised on the 3D VR reconstructions. Owing to their relatively low attenuation, these structures became effectively transparent with the opacity settings applied and were therefore excluded from the rendered 3D representation (Figure 12 and Figure 13). Consequently, their morphology, distribution and anatomical relationships with adjacent organs and vascular structures could not be reliably assessed using the 3D VR images.

Overall, the 3D VR reconstructions complemented the transverse and sagittal MIP series by providing an integrated 3D representation of the principal coelom organs and vascular structures. Thus, whereas the paired MIP reconstructions provided the principal basis for detailed anatomical characterisation using the selected window settings, the 3D VR reconstructions provided complementary information on the 3D topographical relationships of the principal visible coelom structures.

A concise overview of the principal anatomical structures identified within the coelom of an adult male *G. africanus* using contrast-enhanced CT imaging is provided in Supplementary Table S1.

## 4. Discussion

This preliminary contrast-enhanced CT case study describes the appearance and topographical relationships of the coelom in an adult male *G. africanus*, complemented by MIP and 3D VR reconstructions. To the best of our knowledge, no previous study has specifically described the contrast-enhanced CT anatomy of the coelom in this species. Overall, the findings were broadly consistent with established avian anatomical patterns and provide preliminary comparative imaging information for a large Old-World vulture.

The organisation of the avian coelom, including the close association of the viscera with the lung–air-sac system and the relatively limited amount of intracoelom fat, can complicate conventional radiographic interpretation because of anatomical superimposition [12,13,14,15,17,19]. CT reduces this limitation by providing cross-sectional and multiplanar assessment of visceral and vascular structures [16,17,19,21,23,24]. Previous CT studies in raptorial birds provide useful comparative context. In the golden eagle (*Aquila chrysaetos*), intravenous and gastrointestinal contrast media were used, but vascular anatomy was described in less detail, possibly reflecting differences in acquisition and post-processing; that study used contiguous 5-mm transverse images, whereas the present study incorporated transverse and sagittal MIP and 3D VR reconstructions [16]. The common buzzard (*Buteo buteo*) study focused mainly on cranial soft tissues and neurovascular structures [18], while the bald eagle (*Haliaeetus leucocephalus*) study was more methodologically comparable because it also assessed the coelom using contrast-enhanced CT, MPR, MIP, and 3D VR, although with an emphasis on anatomical segmentation and digital visualisation [24]. These methodological differences limit direct interspecific comparison.

The respiratory system contributed substantially to the CT appearance of the coelom. The lungs were positioned dorsally in close association with the vertebral column and ribs, while the air sacs extended among the visceral structures, consistent with established avian anatomy and previous CT studies [12,13,14,15,21,22,23,24]. The very thin walls of normal air sacs could not be consistently visualised, and their configuration may be influenced by anaesthesia, recumbency, respiratory phase, and ventilation. Consequently, the appearance observed in this individual should not be considered an invariant representation of air-sac size or shape.

Intravenous iodinated contrast medium improved differentiation of vascular structures and perfused parenchymal organs and facilitated assessment of their anatomical relationships. The liver, spleen, and kidneys were readily identified, whereas some structures, particularly the pancreas and peripheral neural structures, remained difficult to delineate. The limited conspicuity of the pancreas was likely related to its small size, configuration, and close relationship with the duodenal loop and adjacent soft tissues [12,13,14,15,16,24]. Higher-resolution CT protocols or MRI may permit more detailed evaluation of structures that could not be confidently identified in the present examination.

CT also facilitated assessment of the gastrointestinal tract by reducing anatomical superimposition compared with conventional radiography [17,19,21,23,24]. Nevertheless, luminal gas, fluid, ingesta, distension, and patient positioning may modify the apparent position and relationships of gastrointestinal and adjacent coelomic structures. The anatomical relationships described here should therefore be interpreted in the context of the dorsal recumbency used during image acquisition. Future studies could evaluate whether alternative positioning, including ventral recumbency, improves the separation or visualisation of selected coelomic organs.

Because the examined individual was an adult male, the reproductive anatomy described here cannot be directly extrapolated to females. Reproductive activity may considerably modify the size and topographical relationships of the reproductive tract and adjacent organs, particularly in reproductively active females. Similarly, testicular size may vary with season and reproductive activity. These factors should be considered when interpreting coelom anatomy in individuals of different sex or reproductive status. In reproductively active females, the functional left ovary, located adjacent to the cranial region of the left kidney, may undergo marked follicular enlargement, while the left oviduct becomes substantially developed. These changes may increase the volume occupied by the reproductive tract within the coelom and alter the position and topographical relationships of the kidneys, intestinal loops, and other adjacent coelom structures.

MIP and 3D VR reconstructions provided complementary information to the source CT images. MIP facilitated visualisation of contrast-enhanced vessels and mineralised skeletal structures, although projection of high-attenuation structures may affect apparent spatial relationships [25,26,27]. VR provided an intuitive 3D overview of the major vascular and visceral structures, but its appearance depends on attenuation thresholds, opacity settings, and segmentation strategy [24,25,26,27]. Therefore, transverse and multiplanar source images remain the primary anatomical dataset, with MIP and VR serving as complementary visualisation tools.

Ultrasonography represents a useful complementary technique for real-time evaluation of accessible coelomic soft tissues without ionising radiation [28]. However, limited acoustic windows and gas within the gastrointestinal and respiratory systems may restrict assessment of deeper structures, including the lungs, air sacs, and kidneys [28,29,30]. CT may therefore be particularly useful in cases of complex trauma, skeletal disease, coelomic masses, or lesions in regions where radiographic superimposition or limited ultrasonographic access restricts evaluation [17,27]. In wildlife species, its use should nevertheless be justified by the expected clinical or scientific benefit, considering the requirements for handling, anaesthesia, contrast administration, and radiation exposure [16,27].

The principal limitation of this study is the inclusion of a single adult male, which precludes assessment of inter-individual, sex-related, age-related, and reproductive variation. In addition, no complementary anatomical sections or dissection was performed, preventing direct gross anatomical validation of the CT findings. Physiological and technical factors, including body condition, hydration status, anaesthesia, dorsal recumbency, respiratory phase, contrast-medium dose, and acquisition timing, may also influence the apparent morphology, enhancement, position, and topographical relationships of coelom structures. Accordingly, the attenuation and enhancement patterns observed in this individual should not be interpreted as quantitative normal ranges or species-specific reference data.

To our knowledge, direct CT-based comparisons between *G. africanus* and closely related vulture species are currently lacking. Future studies should therefore include additional *G. africanus* individuals and, where possible, other *Gyps* species using standardised acquisition and contrast-enhancement protocols. Comparisons across sexes, age groups, and reproductive stages may help distinguish general avian anatomical patterns from features shared among large vultures or potentially specific to *G. africanus*.

## 5. Conclusions

Contrast-enhanced CT, complemented by MPR, MIP, and 3D VR reconstructions, enabled detailed evaluation of the principal coelom structures and their spatial relationships in an adult male *G. africanus*. As a preliminary imaging case study, this work demonstrates the potential value of CT for anatomical assessment in *G. africanus* and provides a basis for future comparative and clinical imaging studies. Because only a single individual was examined, the findings should be interpreted as preliminary rather than as species-specific reference data.

## Figures and Tables

**Figure 1:**
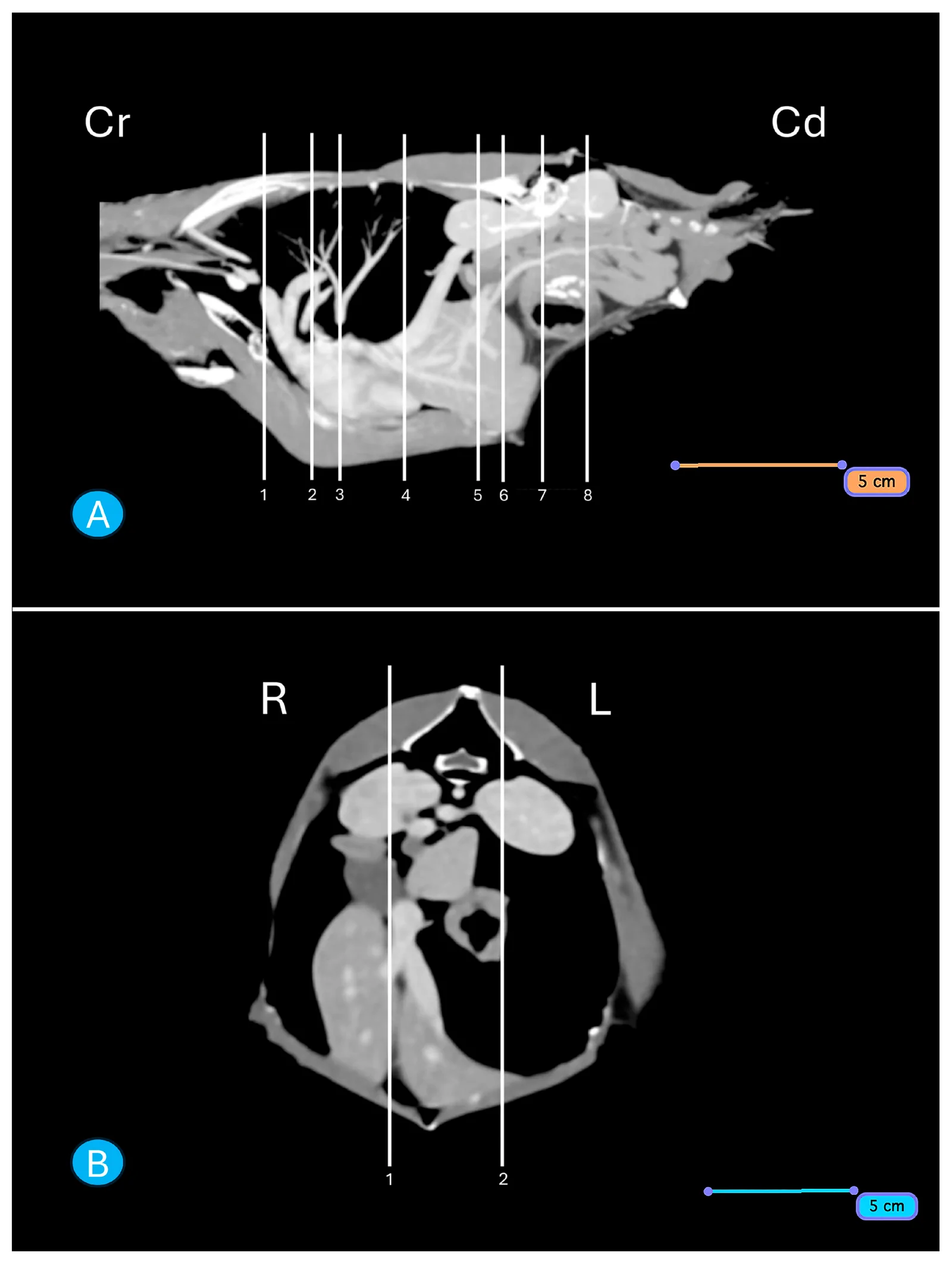
Standard sagittal and transverse reference images showing the approximate locations of the reconstruction planes. (**A**) Sagittal image indicating the craniocaudal levels corresponding to the transverse MIP reconstructions. (**B**) Transverse image indicating the approximate locations of the sagittal MIP planes. Numbers indicate the corresponding reconstruction plane levels. Cr = cranial; Cd = caudal; R = right; L = left. Scale bar = 5 cm.

**Figure 2:**
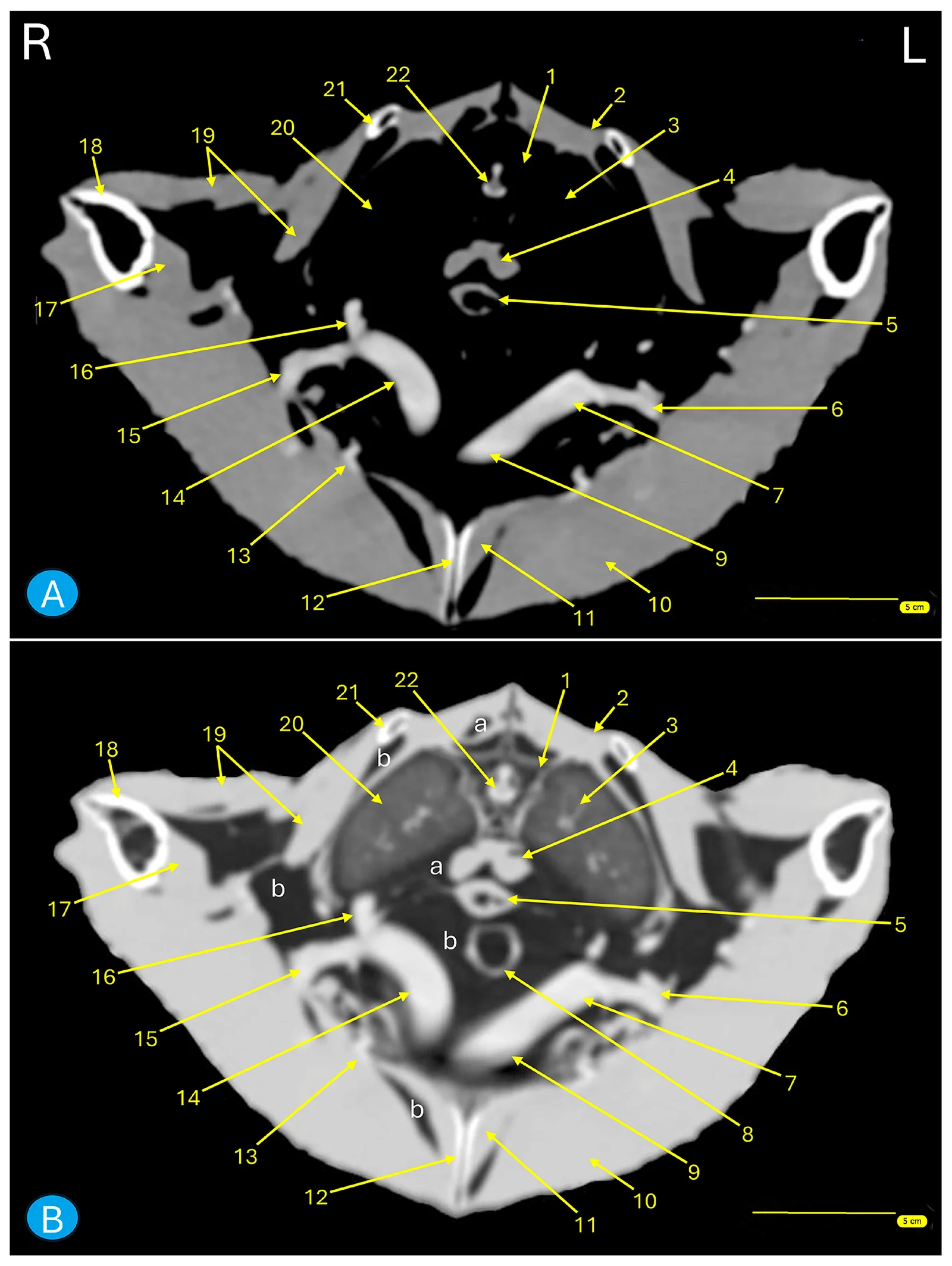
Transverse CT images at the level of the right and left cranial venae cavae (level 1). (**A**) Soft-tissue window and (**B**) lung window. R = right; L = left. 1. Thoracic vertebra; 2. Epaxial muscles; 3. Left lung; 4. Longus colli muscle; 5. Oesophagus; 6. Left brachiocephalic vein; 7. Left cranial vena cava; 8. Trachea (B only); 9. Right atrium; 10. Pectoralis muscle; 11. Supracoracoideus muscle; 12. Sternal carina; 13. Coracoid; 14. Right cranial vena cava; 15. Right brachiocephalic vein; 16. Right external jugular vein and brachiocephalic trunk; 17. Coracobrachialis muscle; 18. Humerus; 19. Cranial and caudal scapulohumeral muscles; 20. Right lung; 21. Scapula; 22. Spinal cord; a. Cervical air sac and associated diverticula (B only); b. Clavicular air sac and associated diverticula (B only). Scale bar = 5 cm.

**Figure 3:**
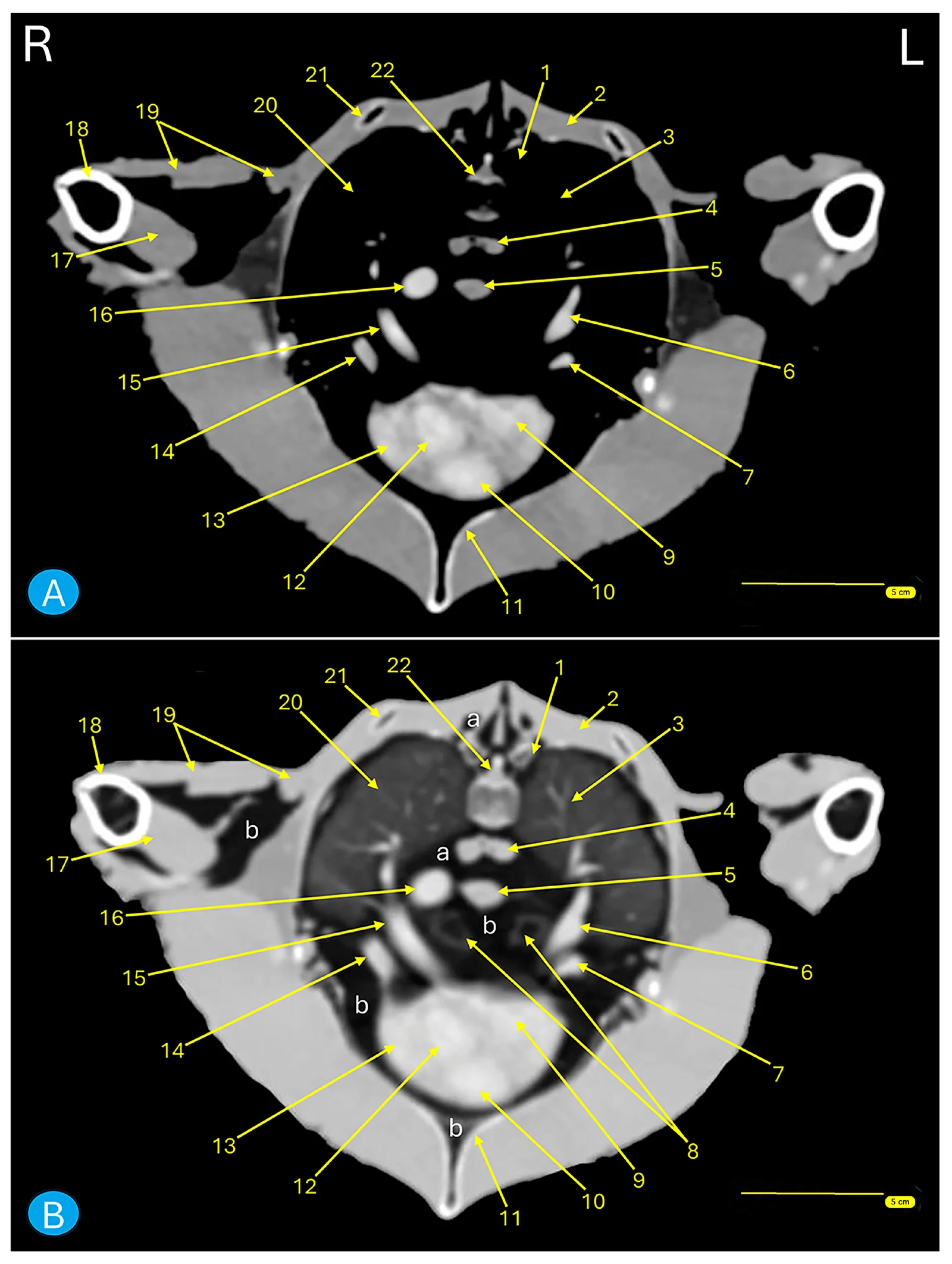
Transverse CT images at the level of the ascending aorta (level 2). (**A**) Soft-tissue window and (**B**) lung window. R = right; L = left. 1. Thoracic vertebra; 2. Epaxial muscles; 3. Left lung; 4. Longus colli muscle; 5. Oesophagus; 6. Left pulmonary artery; 7. Left pulmonary vein; 8. Principal bronchi (B only); 9. Left atrium; 10. Right ventricle; 11. Sternal carina; 12. Ascending aorta; 13. Right atrium; 14. Right pulmonary vein; 15. Right pulmonary artery; 16. Descending aorta; 17. Coracobrachialis muscle; 18. Humerus; 19. Cranial and caudal scapulohumeral muscles; 20. Right lung; 21. Scapula; 22. Spinal cord; a. Cervical air sac and associated diverticula (B only); b. Clavicular air sac and associated diverticula (B only). Scale bar = 5 cm.

**Figure 4:**
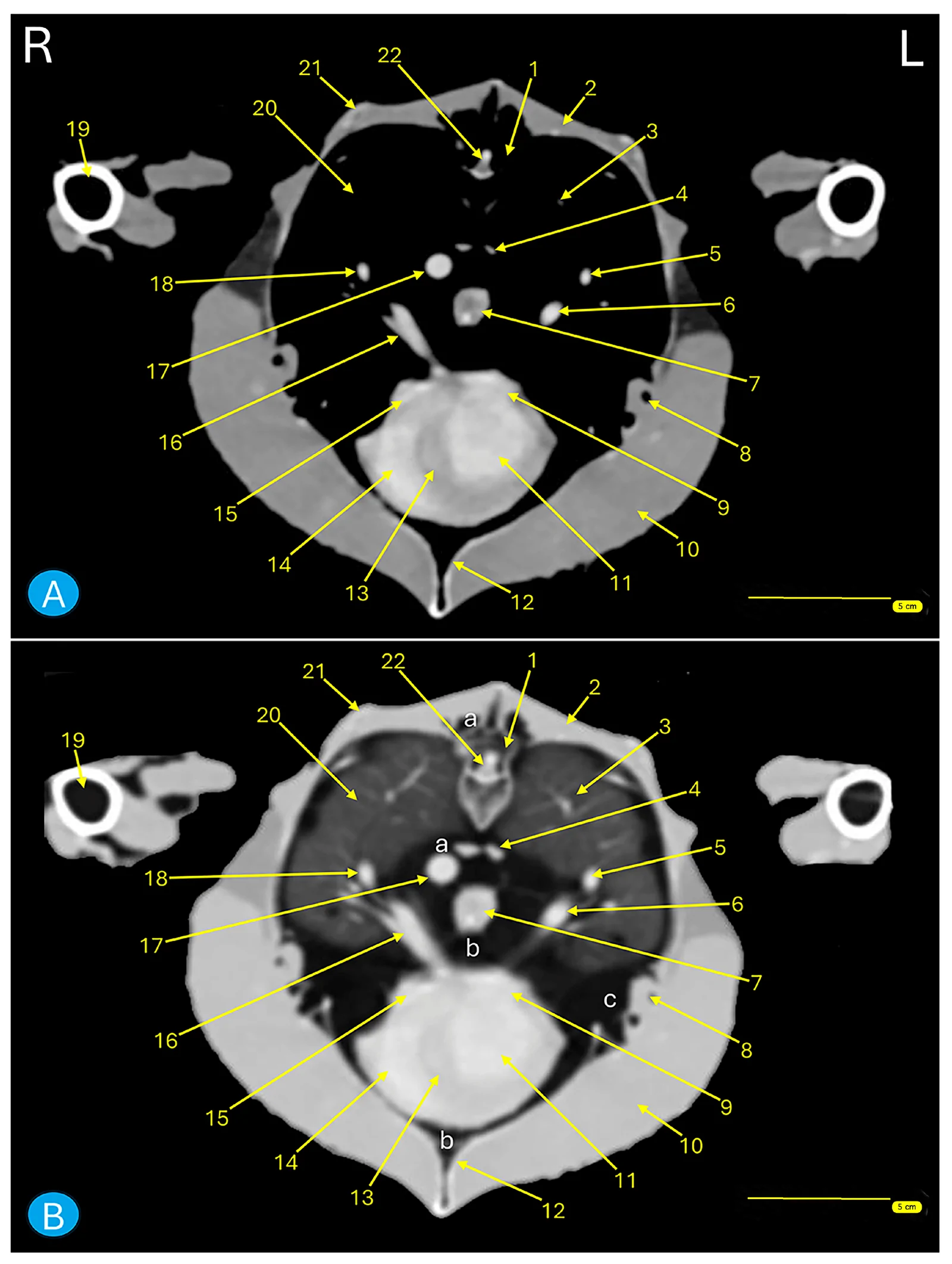
Transverse CT images at the level of the pulmonary trunk (level 3). (**A**) Soft-tissue window and (**B**) lung window. R = right; L = left. 1. Thoracic vertebra; 2. Epaxial muscles; 3. Left lung; 4. Longus colli muscle; 5. Left pulmonary vein; 6. Left pulmonary artery; 7. Oesophagus; 8. Ribs; 9. Left atrium; 10. Pectoralis muscle; 11. Left ventricle; 12. Sternal carina; 13. Interventricular septum; 14. Right ventricle and pulmonary trunk; 15. Caudal vena cava; 16. Right pulmonary artery; 17. Descending aorta; 18. Right pulmonary vein; 19. Humerus; 20. Right lung; 21. Scapula; 22. Spinal cord; a. Cervical air sac and associated diverticula (B only); b. Clavicular air sac and associated diverticula (B only); c. Cranial thoracic air sac (B only). Scale bar = 5 cm.

**Figure 5:**
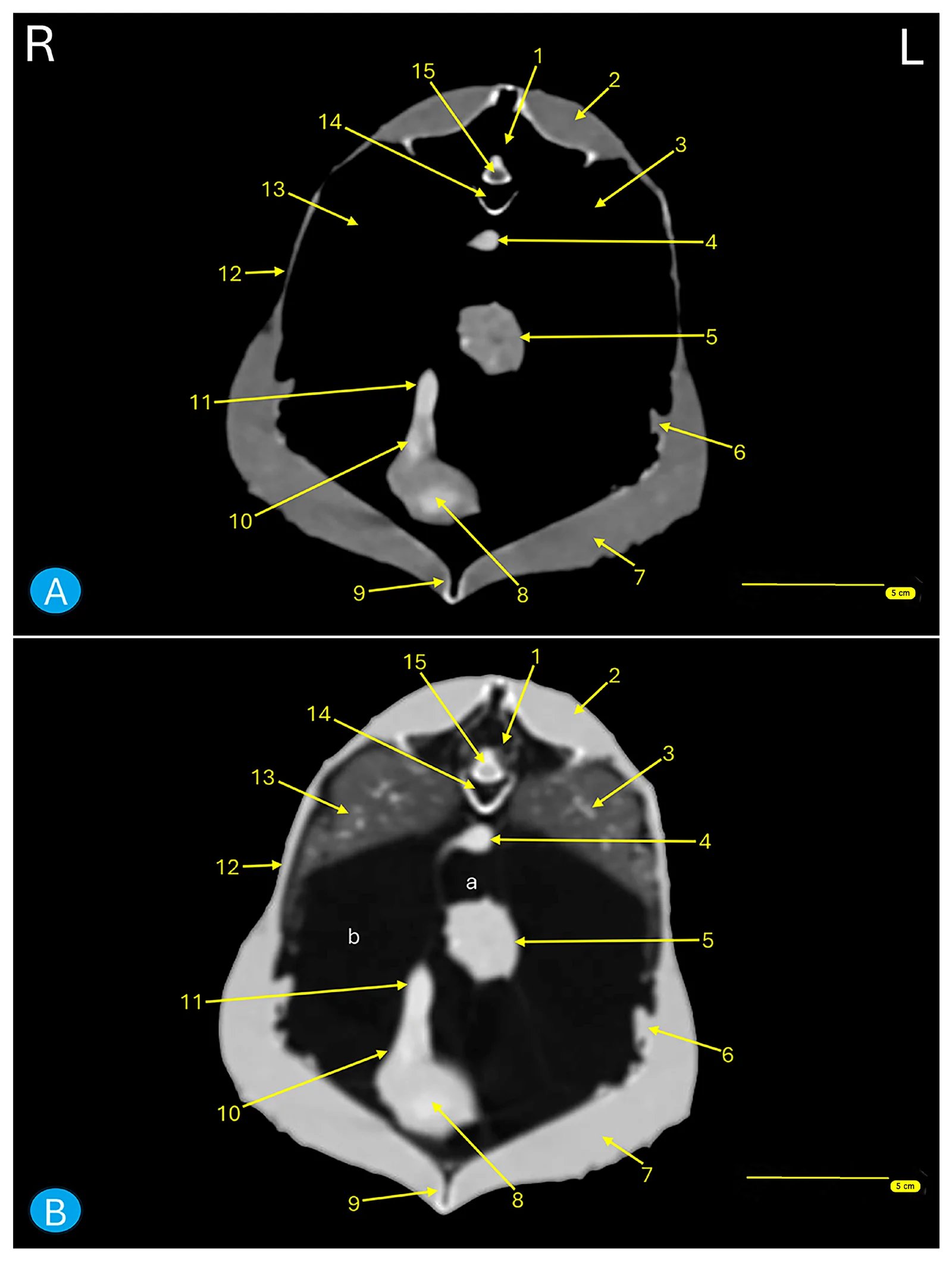
Transverse CT images at the level of the apex of the heart (level 4). (**A**) Soft-tissue window and (**B**) lung window. R = right; L = left. 1. Thoracic vertebral arch; 2. Epaxial muscles; 3. Left lung; 4. Descending aorta; 5. Oesophagus; 6. Ribs; 7. Pectoralis muscle; 8. Apex of the heart; 9. Sternal carina; 10. Right hepatic lobe; 11. Caudal vena cava; 12. Lateral body wall; 13. Right lung; 14. Thoracic vertebral body; 15. Spinal cord; a. Clavicular air sac (B only); b. Cranial thoracic air sac (B only). Scale bar = 5 cm.

**Figure 6:**
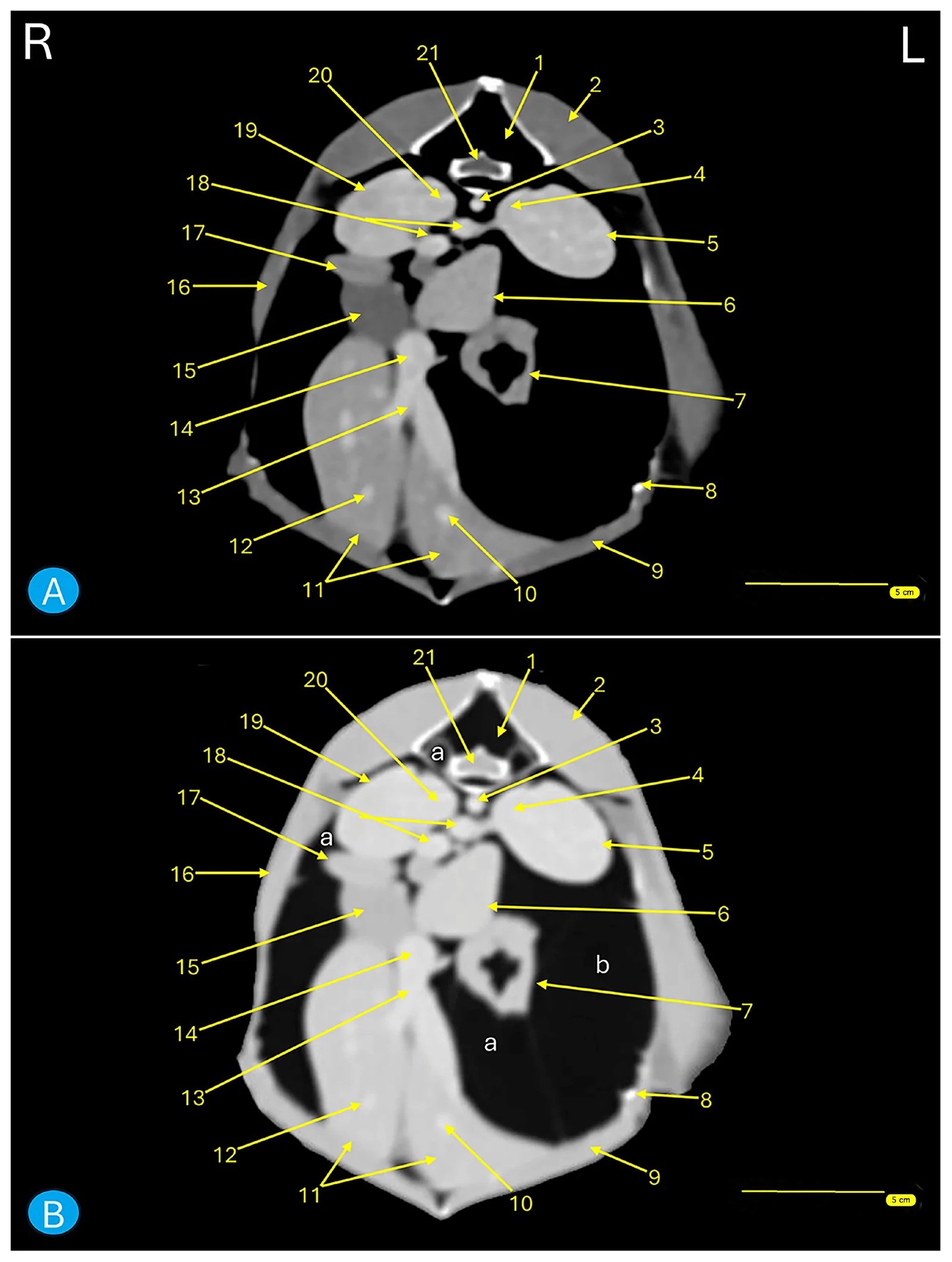
Transverse CT images at the level of the cranial divisions of the kidneys and spleen (level 5). (**A**) Soft-tissue window and (**B**) lung window. R = right; L = left. 1. Synsacrum; 2. Epaxial muscles; 3. Descending aorta; 4. Left testis; 5. Left kidney (cranial division); 6. Spleen; 7. Proventriculus; 8. Ribs; 9. Pectoralis muscle; 10. Left hepatic vein; 11. Right and left hepatic lobes; 12. Right hepatic vein; 13. Right and left hepatic portal veins; 14. Common mesenteric vein; 15. Gallbladder; 16. Lateral body wall; 17. Intestinal loops; 18. Caudal vena cava; 19. Right kidney (cranial division); 20. Right testis; 21. Spinal cord; a. Abdominal air sac and associated recess (B only); b. Caudal thoracic air sac (B only). Scale bar = 5 cm.

**Figure 7:**
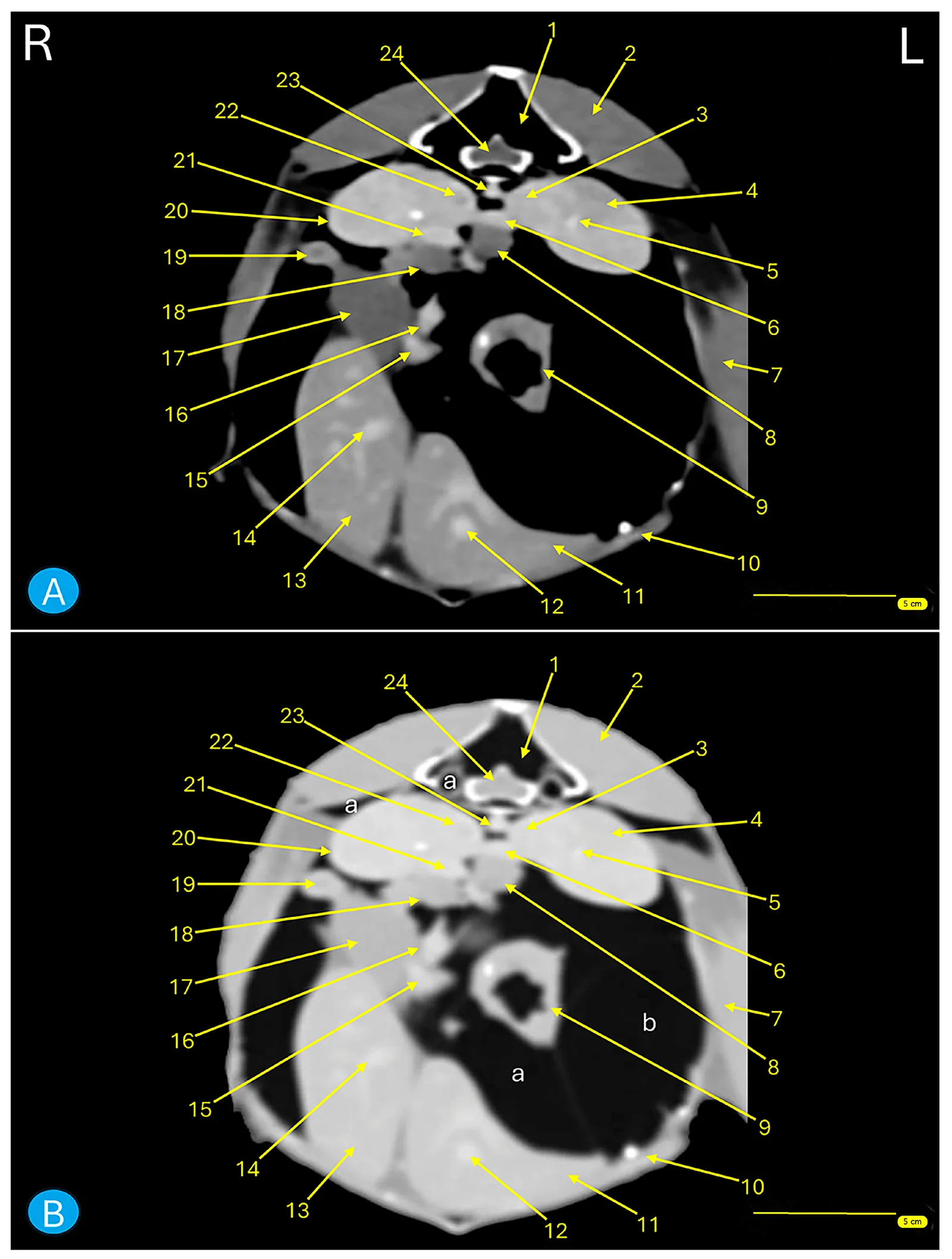
Transverse CT images at the level of the adrenal glands (level 6). (**A**) Soft-tissue window and (**B**) lung window. R = right; L = left. 1. Synsacrum; 2. Epaxial muscles; 3. Left testis; 4. Left kidney (cranial division); 5. Left ureter (renal portion); 6. Left common iliac vein; 7. Lateral body wall; 8. Left adrenal gland; 9. Proventriculus; 10. Ventral body wall; 11. Left hepatic lobe; 12. Left hepatic vein; 13. Right hepatic lobe; 14. Right hepatic vein; 15. Hepatic portal vein; 16. Common mesenteric vein; 17. Gallbladder; 18. Right adrenal gland; 19. Intestinal loop; 20. Right kidney (cranial division); 21. Right common iliac vein; 22. Right testis; 23. Descending aorta; 24. Spinal cord; a. Abdominal air sac and associated recess (B only); b. Caudal thoracic air sac (B only). Scale bar = 5 cm.

**Figure 8:**
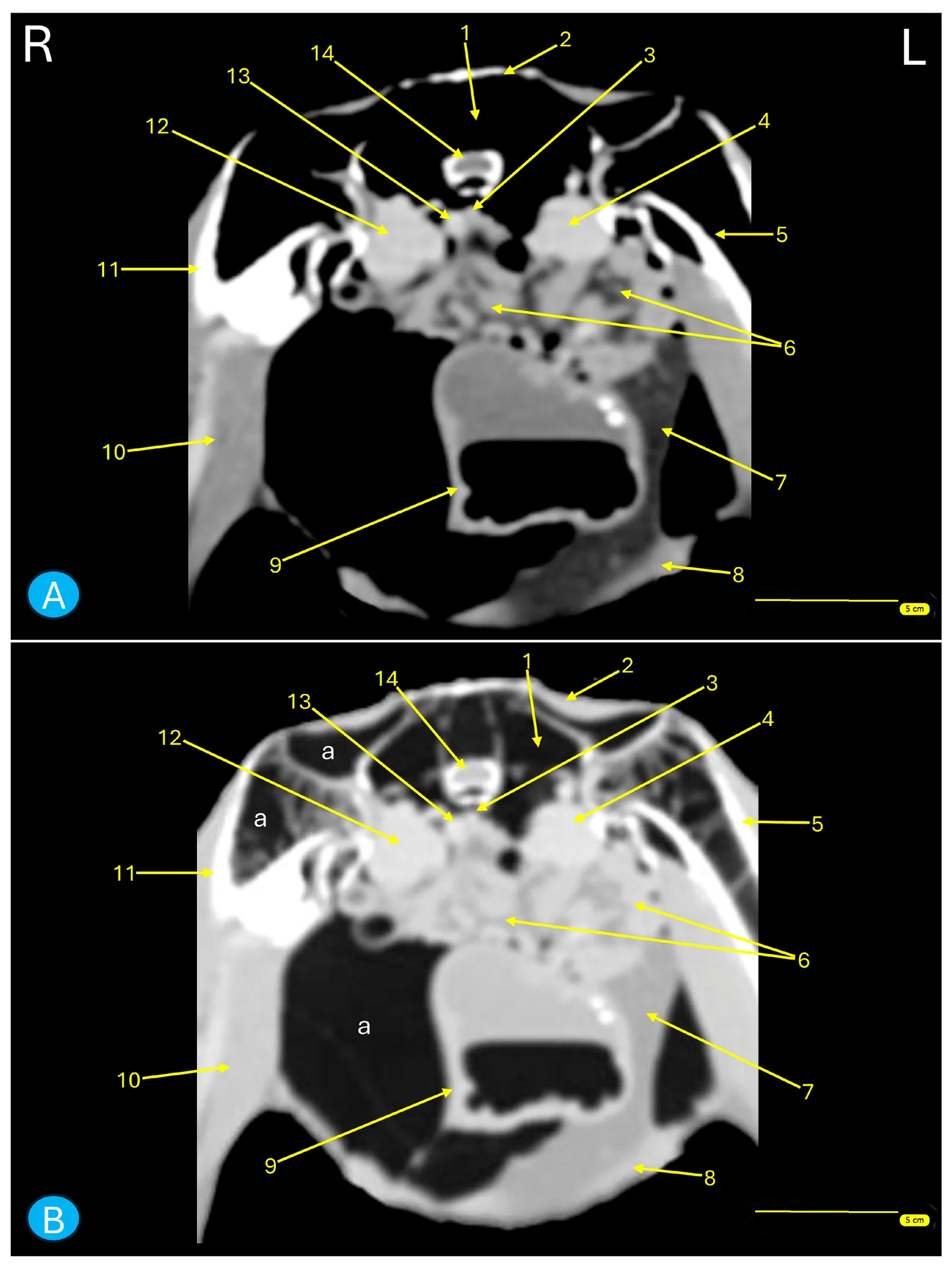
Transverse CT images at the level of the middle divisions of the kidneys (level 7). (**A**) Soft-tissue window and (**B**) lung window. R = right; L = left. 1. Synsacrum; 2. Epaxial muscles; 3. Descending aorta; 4. Left kidney (middle division); 5. Left pelvic limb; 6. Intestinal loops; 7. Coelom fat; 8. Ventral body wall; 9. Ventriculus; 10. Lateral body wall; 11. Right pelvic limb; 12. Right kidney (middle division); 13. Caudal mesenteric vein; 14. Spinal cord; a. Abdominal air sac and associated recess (B only). Scale bar = 5 cm.

**Figure 9:**
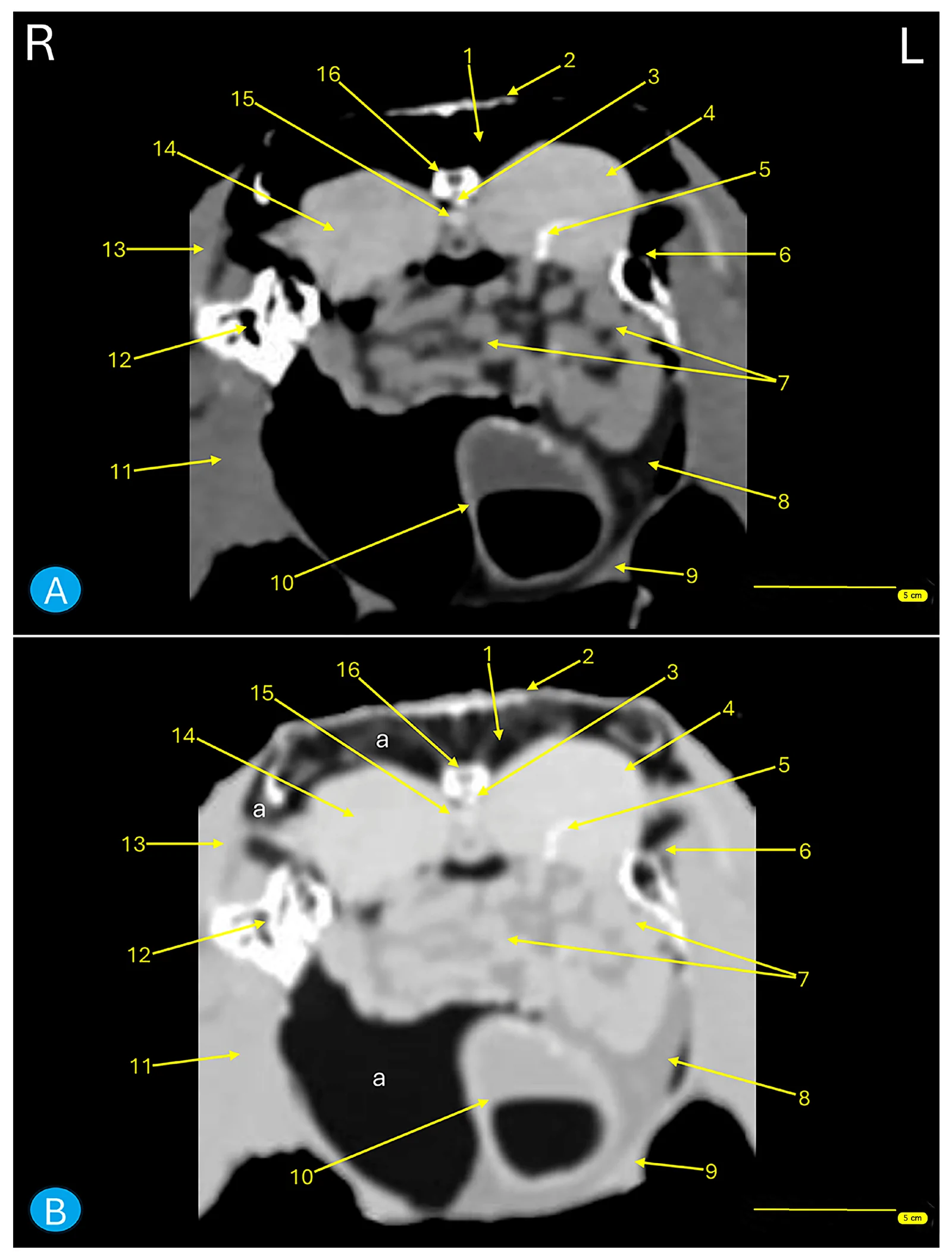
Transverse CT images at the level of the caudal divisions of the kidneys (level 8). (**A**) Soft-tissue window and (**B**) lung window. R = right; L = left. 1. Synsacrum; 2. Epaxial muscles; 3. Descending aorta; 4. Left kidney (caudal division); 5. Left ureter (renal and pelvic portions); 6. Left pelvic limb; 7. Intestinal loops; 8. Coelom fat; 9. Ventral body wall; 10. Ventriculus; 11. Lateral body wall; 12. Right pelvic limb; 13. Pelvic limb muscles; 14. Right kidney (caudal division); 15. Caudal mesenteric vein; 16. Spinal cord; a. Abdominal air sac and associated recess (B only). Scale bar = 5 cm.

**Figure 10:**
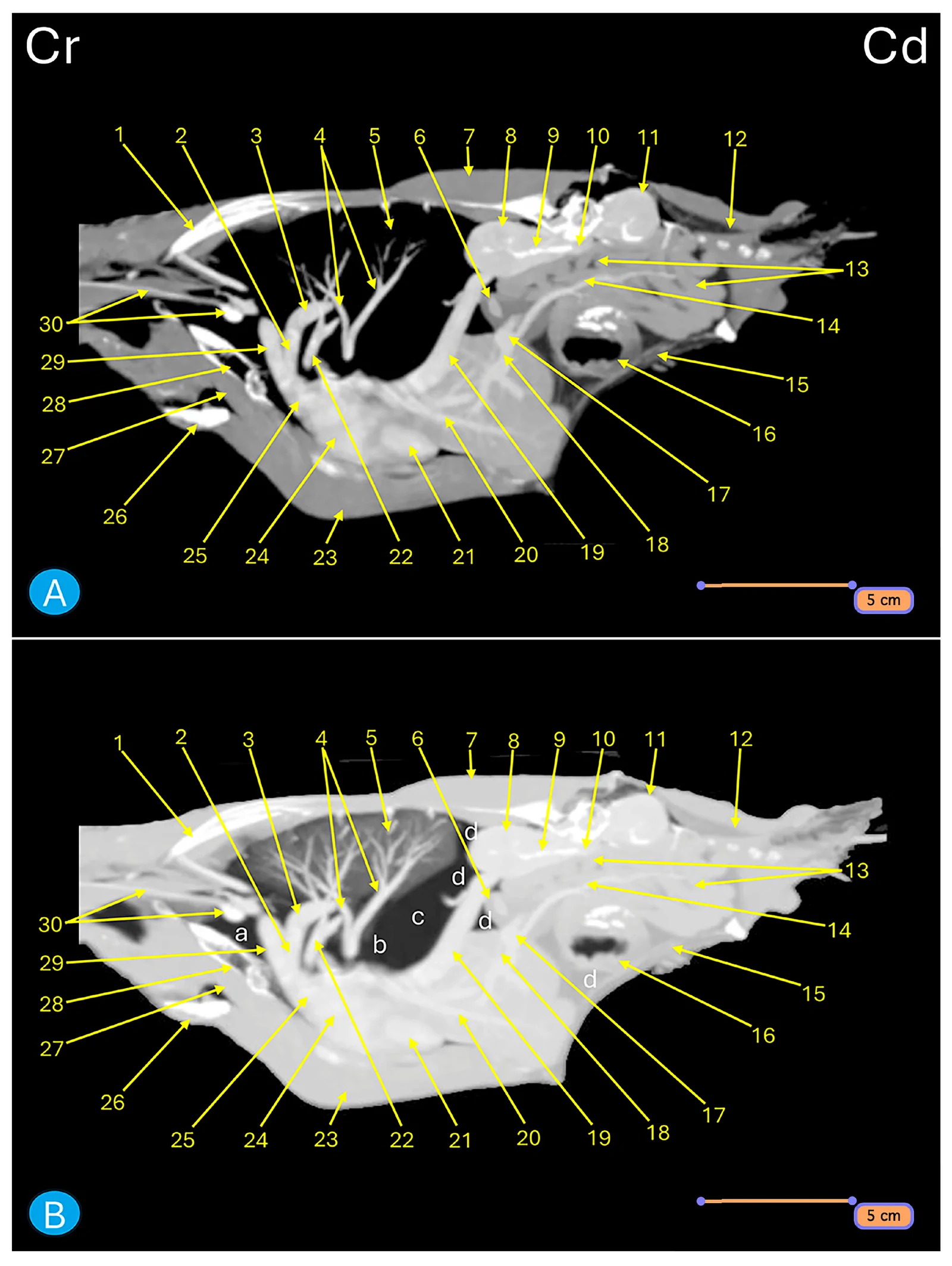
Sagittal CT images at the level of the ascending aorta and aortic arch (level 1). (**A**) Soft-tissue window and (**B**) lung window. Cr = cranial; Cd = caudal. 1. Scapula; 2. Ascending aorta; 3. Aortic arch; 4. Right pulmonary veins; 5. Right lung; 6. Gallbladder and spleen; 7. Epaxial muscles; 8. Right kidney (cranial division); 9. Right ureter (renal portion); 10. Right kidney (middle division); 11. Right kidney (caudal division); 12. Tail; 13. Intestinal loops; 14. Cranial mesenteric vein; 15. Ventral body wall; 16. Ventriculus; 17. Common mesenteric vein; 18. Right hepatic portal vein; 19. Caudal vena cava; 20. Right hepatic lobe and hepatic veins; 21. Left ventricle; 22. Right pulmonary artery; 23. Pectoralis muscle; 24. Right ventricle; 25. Right atrium; 26. Right clavicle; 27. Supracoracoideus muscle; 28. Right coracoid; 29. Right cranial vena cava; 30. Right external jugular and brachiocephalic veins; a. Clavicular air sac and associated diverticula (B only); b. Cranial thoracic air sac (B only); c. Caudal thoracic air sac (B only); d. Abdominal air sac and associated recess (B only). Scale bar = 5 cm.

**Figure 11:**
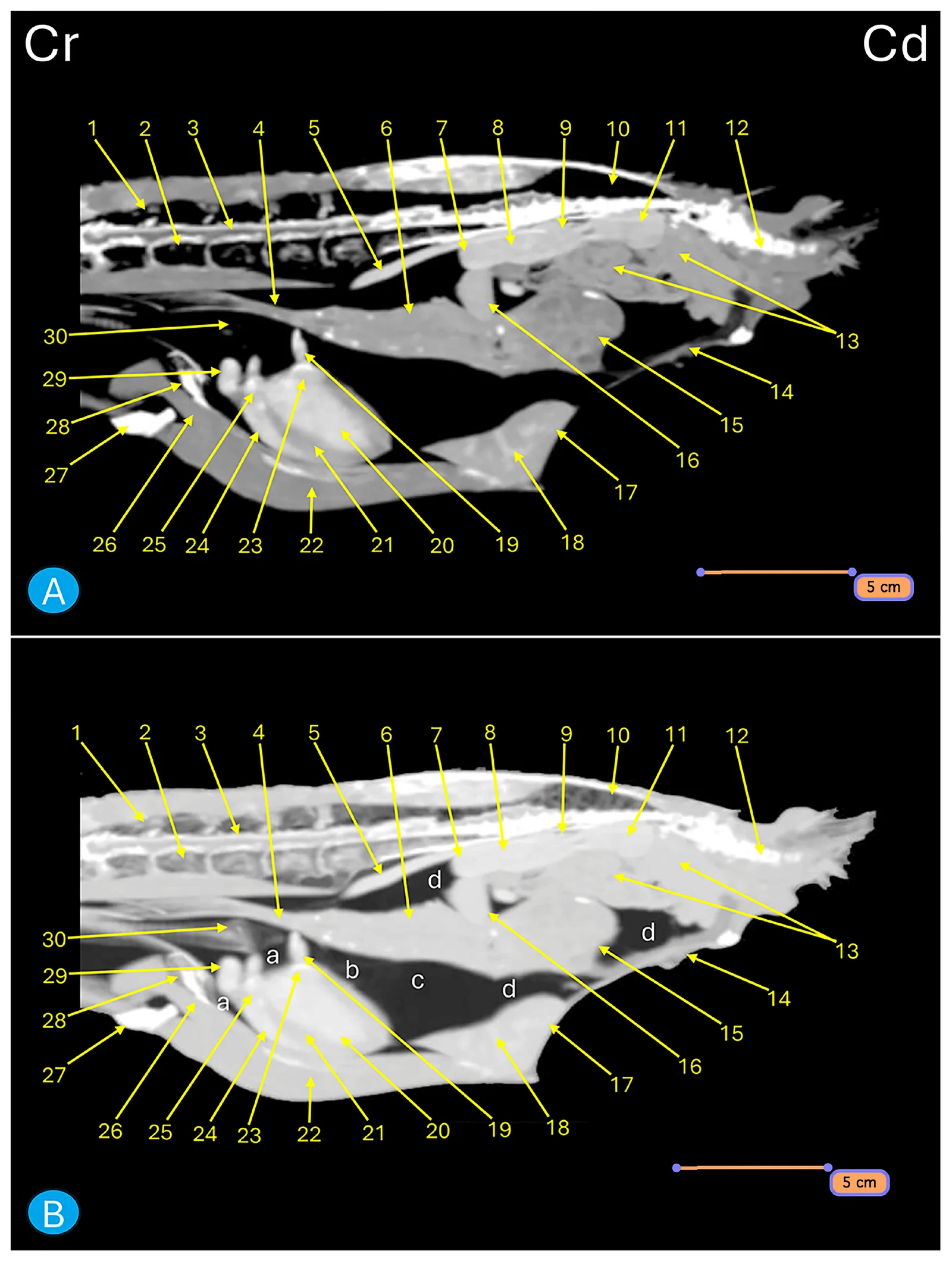
Sagittal CT images at the level of the proventriculus and ventriculus (level 2). (**A**) Soft-tissue window and (**B**) lung window. Cr = cranial; Cd = caudal. 1. Vertebral arch; 2. Vertebral body; 3. Spinal cord; 4. Oesophagus; 5. Descending aorta; 6. Proventriculus; 7. Left testis; 8. Left kidney (cranial division); 9. Left kidney (middle division); 10. Synsacrum; 11. Left kidney (caudal division); 12. Tail; 13. Intestinal loops; 14. Ventral body wall; 15. Ventriculus; 16. Spleen; 17. Left hepatic lobe; 18. Left hepatic veins; 19. Left pulmonary veins; 20. Left ventricle; 21. Interventricular septum; 22. Pectoralis muscle; 23. Left atrium; 24. Right ventricle; 25. Pulmonary trunk; 26. Supracoracoideus muscle; 27. Left clavicle; 28. Left coracoid; 29. Left cranial vena cava; 30. Trachea and syrinx; a. Clavicular air sac and associated diverticula (B only); b. Cranial thoracic air sac (B only); c. Caudal thoracic air sac (B only); d. Abdominal air sac and associated recess (B only). Scale bar = 5 cm.

**Figure 12:**
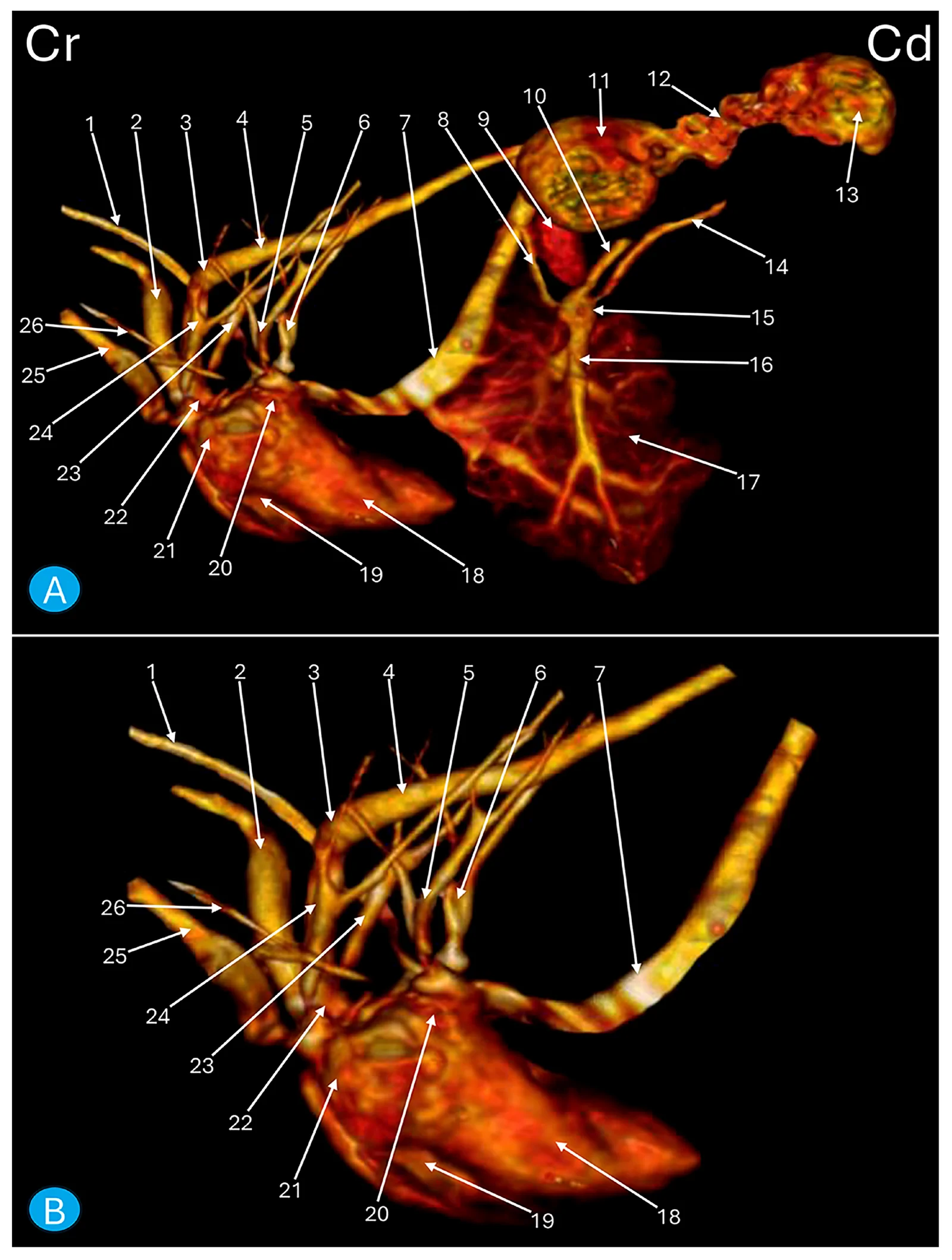
3D VR images of the right lateral surface of the coelom. (**A**) Reconstruction demonstrating the main organs of the coelom. (**B**) Reconstruction illustrating the heart and its major associated vessels. Cr = cranial; Cd = caudal. 1. Left brachiocephalic trunk; 2. Right cranial vena cava; 3. Aortic arch; 4. Descending aorta; 5. Right pulmonary veins; 6. Left pulmonary veins; 7. Caudal vena cava; 8. Splenic and proventricular veins; 9. Spleen; 10. Caudal mesenteric vein; 11. Right kidney (cranial division); 12. Right kidney (middle division); 13. Right kidney (caudal division); 14. Cranial mesenteric vein; 15. Common mesenteric vein; 16. Right hepatic portal vein; 17. Right hepatic lobe and hepatic veins; 18. Left ventricle; 19. Right ventricle; 20. Left atrium; 21. Right atrium; 22. Ascending aorta; 23. Left pulmonary artery; 24. Right pulmonary artery; 25. Left cranial vena cava; 26. Right brachiocephalic trunk. The 3D VR image was used to illustrate spatial relationships rather than for direct morphometric assessment; therefore, no scale bar was included.

**Figure 13:**
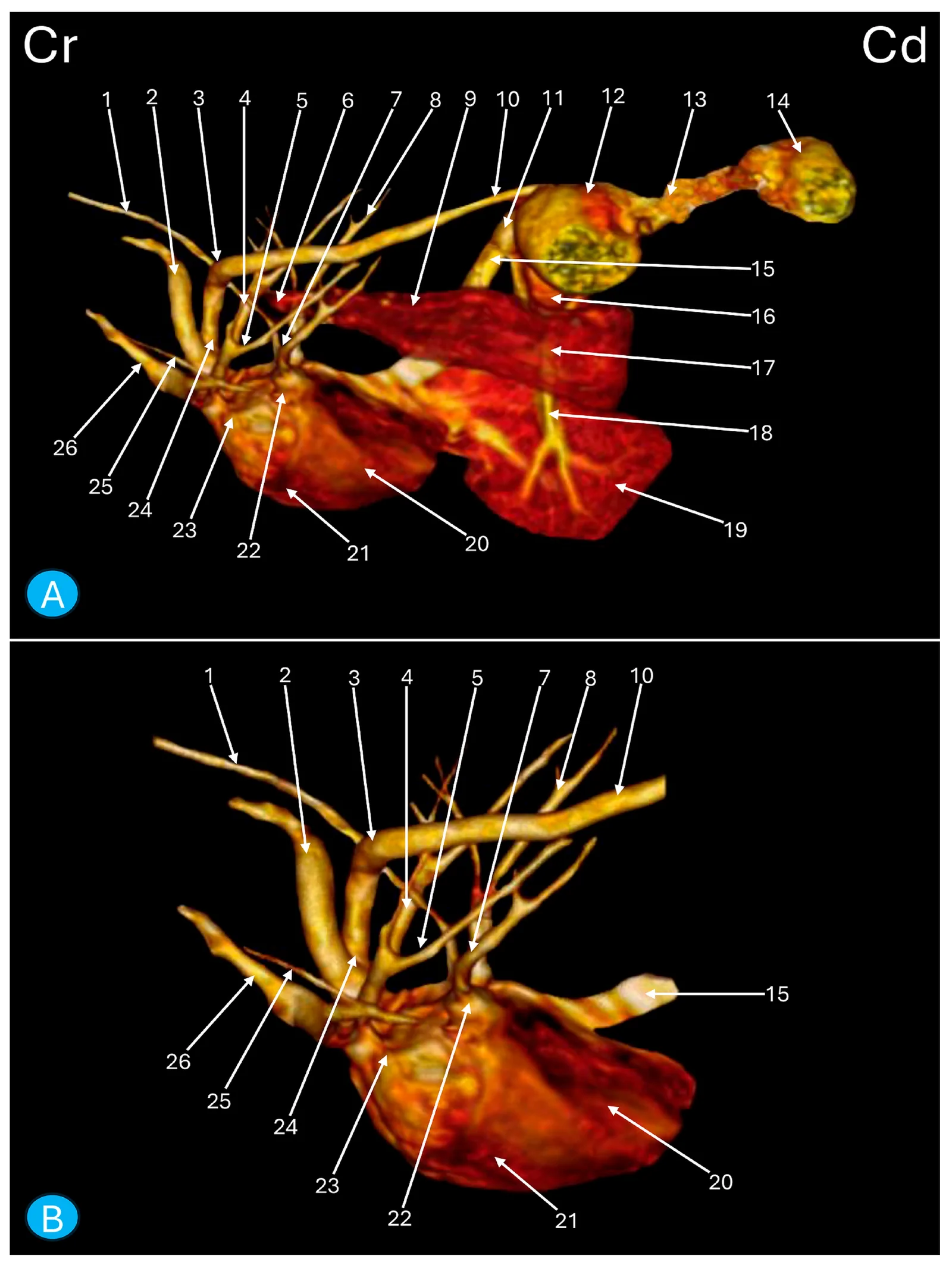
3D VR images of the left lateral surface of the coelom. (**A**) Reconstruction demonstrating the main organs of the coelom. (**B**) Reconstruction illustrating the heart and its major associated vessels. Cr = cranial; Cd = caudal. 1. Right brachiocephalic trunk; 2. Right cranial vena cava; 3. Aortic arch; 4. Right pulmonary artery; 5. Left pulmonary artery; 6. Oesophagus; 7. Left pulmonary veins; 8. Right pulmonary veins; 9. Proventriculus; 10. Descending aorta; 11. Left testis; 12. Left kidney (cranial division); 13. Left kidney (middle division); 14. Left kidney (caudal division); 15. Caudal vena cava; 16. Spleen; 17. Ventriculus; 18. Left hepatic portal vein; 19. Left hepatic lobe and hepatic veins; 20. Left ventricle; 21. Right ventricle; 22. Left atrium; 23. Right atrium; 24. Ascending aorta; 25. Left brachiocephalic trunk; 26. Left cranial vena cava. The 3D VR image was used to illustrate spatial relationships rather than for direct morphometric assessment; therefore, no scale bar was included.

## Data Availability

The DICOM images presented and analysed in this study are available upon reasonable request from mario.encinoso@ulpgc.es and alberto.arencibia@ulpgc.es.

## Supplementary Materials

The following supporting information can be downloaded at https://www.mdpi.com/article/10.3390/ani16193079/s1. Table S1. Characterisation of coelom structures of an adult male African white-backed vulture (*Gyps africanus*) using contrast-enhanced CT.

## Abbreviations

The following abbreviations are used in this manuscript:

CT	Computed tomography
DICOM	Digital imaging and communications in medicine
HU	Hounsfield unit
MIP	Maximum intensity projection
MPR	Multiplanar reconstruction
VR	Volume rendering
3D	Three-dimensional
