# Supplementary material for: Preliminary Contrast-Enhanced CT Imaging of the Coelom in an Adult Male African White-Backed Vulture (Gyps africanus): A Case Study Using Maximum Intensity Projection and Three-Dimensional Volume Rendering
Source: Animals (Basel). 2026 Sep 30;16(19):3079. doi: 10.3390/ani16193079 (PMC13663397; doi:10.3390/ani16193079)
Supplement: Supplementary file 1 [file animals-16-03079-s001.zip › animals-4574445-supplementary.pdf]

Supplementary table

Table S1. Characterisation of coelom structures of an adult male African white-backed vulture (*Gyps africanus*) using contrast-enhanced CT.

ANATOMICAL STRUCTURE	CT SOFT-TISSUE WINDOW	CT LUNG WINDOW	3D VR
**Cortical bone**	Osseous cortices showed very high attenuation and clearly demarcated external boundaries.	Cortical components remained intensely hyperattenuating, with crisp, readily distinguishable contours.	Not visualised.
**Bone marrow**	Marrow cavities displayed lower attenuation than the surrounding cortical bone.	Marrow detail was poorly conspicuous and partly masked by neighbouring air-containing structures.	Not visualised.
**Muscles**	Muscular tissues appeared as moderately attenuating soft-tissue structures.	Differentiation of individual muscles from adjacent soft tissues was limited.	Not visualised.
**Heart**	The cardiac silhouette showed moderately increased attenuation and distinct margins.	Cardiac boundaries were more conspicuous because of the marked contrast with the surrounding aerated pulmonary tissue.	The heart was readily identifiable as a centrally positioned structure.

**Blood vessels**	Vascular structures appeared hyperattenuating and could be followed along well-defined margins.	Markedly hyperattenuating branching vessels, clearly differentiated from the lungs and air sacs.	3D reconstruction provided clear depiction of the vascular architecture and its major ramifications.
**Trachea**	Very low-attenuation air-filled tube with a thin soft-tissue wall.	ts air-filled lumen was highly conspicuous, whereas the surrounding wall was only faintly perceptible.	Not visualised.
**Syrinx**	The syrinx presented predominantly soft-tissue attenuation corresponding to its cartilaginous and membranous elements.	It was recognised by the contrast between its very low-attenuation air column and the thin surrounding soft-tissue components.	Not visualised.
**Lungs**	Pulmonary tissue was characterised by markedly reduced attenuation.	Pulmonary architecture became more readily distinguishable and displayed a heterogeneous attenuation pattern.	Not visualised.
**Air sacs**	Air sacs appeared as gas-filled spaces with extremely low attenuation.	These air-containing compartments were clearly separable from the adjacent pulmonary parenchyma.	Not visualised.
**Oesophagus**	The oesophagus was observed as a mildly hyperattenuating tubular structure with discernible boundaries.	A narrow tubular course could be followed more readily because of its slightly increased attenuation.	Only a limited portion of the tubular structure could be delineated.

**Glandular stomach (Proventriculus)**	The proventriculus appeared as a narrow tubular organ with thin walls, relatively uniform contrast enhancement and distinct margins.	Its intermediate attenuation resulted in limited differentiation from neighbouring soft tissues.	The organ was only partially outlined as a tubular structure.
**Muscular stomach (Ventriculus)**	The ventriculus was recognised as a prominent muscular organ exhibiting relatively uniform, moderate attenuation.	Delineation from adjacent tissues was reduced because of the limited inherent soft-tissue contrast.	A rounded muscular structure could be recognised with moderate definition.
**Liver**	The hepatic parenchyma was homogeneous and showed mildly reduced attenuation.	Evaluation of internal hepatic detail was restricted by poor soft-tissue discrimination.	The liver was clearly represented as a bilobed structure occupying the cranial portion of the coelom.
**Gallbladder**	A low-attenuation, fluid-containing structure surrounded by a thin wall was identified.	Its boundaries were difficult to distinguish because of insufficient soft-tissue contrast.	Not visualised.
**Spleen**	The spleen appeared as a small, oval and well-circumscribed structure with mildly increased attenuation.	Splenic margins were poorly differentiated from surrounding tissues.	A small structure associated with the gastric region could be identified.
**Pancreas**	The pancreas could not be confidently identified.	The pancreas was not distinguishable.	No pancreatic structure was evident.
**Intestines**	Intestinal loops were thin walled and contained variable proportions of intraluminal gas and fluid.	Individual intestinal segments were difficult to evaluate because of reduced soft-tissue contrast.	Multiple convoluted tubular structures occupied the middle and caudal portions of the coelom.

Table S1 (Cont.)

ANATOMICAL STRUCTURE	CT SOFT-TISSUE WINDOW	CT LUNG WINDOW	3D VR
**Kidneys**	Both kidneys appeared as lobulated, moderately hyperattenuating structures situated in the retroperitoneal position.	Renal tissue remained relatively hyperattenuating, although internal soft-tissue differentiation was limited.	The kidneys were depicted as paired elongated structures.
**Adrenal glands**	Small, poorly conspicuous soft-tissue structures were identified immediately cranial to the kidneys.	Reliable identification was hindered by the low contrast between adjacent soft tissues.	Not visualised.
**Ureters**	The ureters were variably detectable following contrast administration as paired, delicate tubular structures extending caudally from the kidneys.	Their course was only faintly distinguishable, and the tubular margins remained poorly defined.	Not visualised.
**Testes**	The testes were identified as paired, small, ovoid soft-tissue structures positioned cranial to the kidneys and showing mildly increased attenuation.	Testicular margins were difficult to distinguish because of the limited contrast with adjacent soft tissues.	Not visualised.
**Nerves**	Not visualised.	Not visualised.	Not visualised.
